# CancerSupportSource®-15+: development and evaluation of a short form of a distress screening program for cancer patients and survivors

**DOI:** 10.1007/s00520-021-05988-2

**Published:** 2021-01-14

**Authors:** Alexandra K. Zaleta, Shauna McManus, Erica E. Fortune, Branlyn W. DeRosa, Joanne S. Buzaglo, Julie S. Olson, Sara Goldberger, Melissa F. Miller

**Affiliations:** 1 Cancer Support Community, Research and Training Institute, 520 Walnut Street, Suite 1170, Philadelphia, PA 19106 USA; 2 Present affiliation: Children’s Hospital of Philadelphia, Division of Oncology, Philadelphia, PA USA; 3 Present affiliation: ConcertAI, Boston, MA USA; 4 Cancer Support Community, New York, NY USA

**Keywords:** Cancer, Distress, Screening, Survivorship, Depression, Anxiety

## Abstract

**Purpose:**

CancerSupportSource® (CSS) is a distress screening program implemented at community-based organizations and hospitals nationwide. The 25-item CSS assesses distress across five domains, with capacity to screen for clinically significant depression and anxiety. This study examined psychometric properties of a shortened form to enhance screening opportunities when staff or patient burden considerations are significant.

**Methods:**

Development and validation were completed in multiple phases. Item reduction decisions were made with 1436 cancer patients by assessing external/internal item quality and judging theoretical and practical implications of items. Pearson correlations and confirmatory factor analysis were conducted on a separate sample of 957 patients to corroborate psychometric properties and dimensionality of the shortened scale. Nonparametric receiver operating characteristic (ROC) curve analyses determined scoring thresholds for depression and anxiety risk scales.

**Results:**

Scale refinement resulted in a 15-item short form plus one screening item assessing tobacco and substance use (CSS-15+). At least two items from each CSS domain were retained to preserve multidimensionality. In confirmatory analysis, the model explained 59% of the variance and demonstrated good fit. Correlation between CSS-15+ and 25-item CSS was 0.99, *p* < 0.001. Sensitivity of 2-item depression and 2-item anxiety risk scales in the confirmatory sample were 0.82 and 0.83, respectively.

**Conclusions:**

CSS-15+ is a brief, reliable, and valid multidimensional measure of distress. The measure retained excellent internal consistency (*α* = 0.94) and a stable factor structure. CSS-15+ is a practical and efficient screening tool for distress and risk for depression and anxiety among cancer patients and survivors, particularly in community-based settings.

## Background

Cancer can impact all aspects of patients’ lives, including physical, emotional, and practical consequences [1]. Many patients in the community experience significant levels of distress, with estimates ranging from 25 to 50% [2]. The term distress is not limited to mental health issues, rather it is defined as, “a multifactorial unpleasant experience of a psychological (i.e., cognitive, behavioral, emotional), social, spiritual, and/or physical nature that may interfere with one’s ability to cope effectively with cancer, its physical symptoms, and its treatment” [3]. Given the prevalence of distress in the cancer experience, as well as its impact on physical health and cancer outcomes [4, 5], screening for distress has become an integral part of caring for the whole patient in cancer care [1]. Notably, distress screening is now mandated for cancer centers to achieve continuing accreditation by the American College of Surgeons Commission on Cancer as well as to meet requirements for American Society of Clinical Oncology Quality Oncology Practice Initiative (QOPI®) standards, Oncology Care Model (OCM) quality measures, and National Comprehensive Cancer Network (NCCN) distress screening guidelines [3, 6–8].

To meet the demand for integrated and effective distress screening in cancer care settings, there is a well-established need for reliable and valid methods of screening and referring cancer patients for distress management [1, 9, 10]. There is also increased focus on the importance of understanding the feasibility of, and barriers to, implementing distress screening programs in real-world practice settings [10–12]. Developing distress screening tools that are both psychometrically sound and feasible for patients and providers is particularly important for uptake in community-based cancer care settings, in which the need for assessment and demand for resources may be substantial relative to the number of trained support staff.

In response to this critical need, Cancer Support Community (CSC) developed *CancerSupportSource*® (CSS), a reliable, valid, multidimensional distress screening program with the capacity to identify those at risk for clinically significant levels of anxiety and depression. CSS is web-based and, in full implementation, includes a follow-up and referral program to assist community-based cancer centers in meeting distress screening accreditation standards linked to patient-centered cancer care [13].

Development and comprehensive psychometric validation of CSS has been previously described [14]. CSS assesses distress over five key domains (*emotional well-being*, *symptom burden and impact*, *body image and healthy lifestyle*, *health care team communication*, *and relationships and intimacy*) and also includes an item assessing tobacco and substance use. The five-factor model is replicable and the risk subscales demonstrated high sensitivity and adequate specificity [14].

CSS is currently implemented across CSC’s network of community-based cancer support affiliates as well as in oncology practices and hospital cancer centers nationwide. In community-based care settings, there is a need for flexibility in assessment while still maintaining instrument fidelity. The creation of shorter scales is a well-established practice when an abbreviated measure is needed due to variety of practical considerations, including being used for a different purpose (e.g., in clinical trials versus longer clinical evaluation) [15], or in different settings (e.g., fast-paced medical clinic versus explanatory research) [16]. Availability of a concise, abbreviated version of CSS may enhance opportunities for distress screening in care settings where time and patient burden considerations are significant.

The aims of the current study were to (1) develop a shortened version of CSS and (2) to examine the psychometric properties of the shortened scale.

## Methods and results

An abbreviated version of CSS was guided by best-practice guidelines, including using internal, external, and judgmental item characteristics to inform item retention decisions [17]. Development and validation were completed in two phases with two separate samples of participants: (1) scale reduction and (2) corroboration of psychometric properties and dimensionality.

### Phase I: scale reduction

#### Participants

Participants for scale-reduction decisions were the same as in the CSS validation study [14]. Data were collected through the Cancer Support Community’s Cancer Experience Registry (CER), an online, community-based research initiative examining the social and emotional impact of cancer. Participants in the validation study included 1436 cancer patients and survivors (Table 1) who participated in the CER survey from March 2013 to December 2016, were 18 years or older, lived in the USA, completed at least 22 of the 25 distress items, and also completed at least one comparative validation measure.

**Table 1:** Descriptive characteristics of the sample

		Phase I data (*N* = 1436)		Phase II data (*N* = 957)	
		*M/n*	SD (%)	*M/n*	SD (%)
Age^†^		58.4	11.1	58.25	12.23
Gender	Female	1035	72%	642	67%
	Male	401	28%	298	31%
Race	White	1291	90%	797	83%
	African American	64	4%	69	7%
	Asian	18	1%	9	1%
	American Indian or Alaskan Native	5	< 1%	3	< 1%
	Native Hawaiian or Pacific Islander	4	< 1%	1	< 1%
	Multiple races	25	2%	18	2%
Hispanic or Latino/a		40	3%	52	5%
Education	No college	196	14%	167	18%
	Some college	293	20%	235	25%
	College degree	534	37%	316	33%
	Graduate or professional degree	402	28%	215	23%
Annual income	< $20 K	108	8%	121	13%
	$20–39 K	207	14%	134	14%
	$40–59 K	181	13%	112	12%
	$60–79 K	145	10%	98	10%
	$80–99 K	136	9%	84	9%
	$100 K+	300	21%	174	18%
	Prefer not to share	283	20%	198	21%
Employment	Full-time	450	31%	288	30%
	Part-time	138	10%	82	9%
	Retired	447	31%	287	30%
	Disability	256	18%	194	20%
	Unemployed	110	8%	69	7%
Cancer diagnosis (most recent)	Breast	504	35%	297	31%
	Hematologic	497	35%	100	10%
	Lung	65	5%	91	10%
	Prostate	54	4%	150	16%
	Ovarian	50	3%	36	4%
	Colorectal	44	3%	46	5%
	Melanoma	34	2%	22	2%
	Head and Neck	20	1%	29	3%
	Endometrial	18	1%	20	2%
	Sarcoma	11	1%	16	2%
	Stomach	8	1%	14	2%
	Other^‡^	131	9%	128	13%
Years since diagnosis^†^		4.6	5.3	4.3	6.1
Stage	0	93	6%	55	6%
	I	295	21%	186	20%
	II	319	22%	174	18%
	III	319	22%	174	18%
	IV	194	14%	132	14%
	Other	45	3%	26	3%
	Do not know	154	11%	131	14%
Treatment history	Current chemotherapy	301	21%	228	24%
	Current radiation therapy	62	4%	166	17%
	Current hormonal therapy	217	15%	175	18%
	Ever chemotherapy	1029	72%	562	59%
	Ever radiation therapy	629	44%	433	45%
	Ever hormonal therapy	309	22%	236	25%
	Past surgery	878	61%	631	66%

^†^Subsample sizes for Phase 1: age (*n =* 1309), years since diagnosis (*n =* 1430) and Phase 2: age (*n =* 906), years since diagnosis (*n =* 871)

^‡^Other cancer diagnoses included endometrial, cervical, pancreatic, bladder, esophageal, kidney/renal cell, brain, testicular, and anal, among others

aside from age and years since diagnosis, the reported proportions above are calculated out of the total sample Ns: Phase 1 (*N =* 1436) and Phase 2: (*N =* 957). Percentages may not total 100% due to incomplete or missing data

Ethical and Independent Review Services (E&I, Independence, MO) served as the IRB of record. All procedures performed in studies involving human participants were in accordance with the ethical standards of the institutional research committee and with the 1964 Helsinki declaration and its later amendments or comparable ethical standards. Informed consent was obtained from all individual participants included in the CER.

#### Measures

Socio-demographics and clinical history: participants provided demographic and clinical background information (age, gender, race, ethnicity, education, employment status, household income, self-reported cancer diagnosis, stage at diagnosis, time since diagnosis, and types of treatments received.

CancerSupportSource: cancer-related distress was assessed using the 25-item version of CSS (CSS-25). Patients rated their level of concern (0 *not at all*, 1 *slightly*, 2 *moderately*, 3 *seriously*, and 4 *very seriously*) for each item; request for follow-up services was not assessed for the current study.

PROMIS: participant self-reported symptoms and functioning were examined using the Patient-Reported Outcomes Measurement Information System-29 (PROMIS-29 v2.0) [18]. Five domains assess symptoms with higher scores corresponding to worse symptomology (depression, anxiety, pain interference, fatigue, and sleep disturbance) and two domains assess function with lower scores corresponding to worse functioning (physical function, ability to participate in social roles and activities). Participants rate each item with reference to the past seven days; function scales have no timeframe specified. Scale scores are converted to standardized *T* scores (mean = 50, SD = 10); normative reference groups are the U.S. general population, except sleep disturbance, where comparisons are to a mix of the U.S. population and people with chronic illness.

#### Analysis

Data analysis was conducted using IBM SPSS Statistics 24.0 [19] and R 3.4.0 [20], with GPArotation [21] and psych [22] R packages. For each item in CSS-25, external item quality, internal item quality, and judgmental item quality were assessed independently by authors; these three indices were then reconciled in a series of consensus meetings to reduce the scale to a shortened version:

External item quality: external item quality was assessed by examining Pearson correlations between CSS-25 items and PROMIS-29 subscales.

Internal item quality: internal item quality was assessed by evaluating item discrimination indices, inter-scale and inter-factor correlations, factor loadings and structure, and item communalities from an exploratory factor analysis of CSS-25. The exploratory factor analysis, which was previously published in the validation of CSS-25 [14], was conducted with direct oblique rotation and principal axis factoring (PAF) extraction.

Judgmental item quality: judgmental item quality involved the ranking and prioritization of all CSS-25 items by CSS-25 developers, accounting for theoretical and practical implications, with particular attention to content validity.

#### Results

Participant socio-demographics*:* participants were predominantly female (72%), White (90%), and completed a college degree (65%). The average age was 58 years (range = 19 to 87), and average time since initial cancer diagnosis was 4.6 years (range = < 1 to 52). The most commonly represented diagnoses included breast cancer (35%) and hematologic cancers (35%; Table 1).

External item quality: scores for each item were correlated with standardized *T* scores for each PROMIS-29 subscale (range: 0.49 to 0.70). Stronger correlations were exhibited between items measuring similar constructs as PROMIS subscales; e.g., the two items that make up the depression risk screening subscale and the two items that make up the anxiety risk screening subscale were highly correlated with the PROMIS depression and anxiety subscales, respectively. Correlations for each subscale were sorted from high to low within each CSS-25 factor.

Internal item quality: the first CSS-25 factor includes 8 items assessing emotional concerns; the depression risk screening items and the anxiety risk screening items were the highest loading items within this factor. Corrected item-factor correlations ranged from 0.60 to 0.82. The highest loading items within the 8-item symptom burden and impact domain included items assessing pain/physical discomfort, functional ability, and fatigue; corrected item-factor correlations ranged from 0.51 to 0.81. Within the 4-item domain assessing body and healthy lifestyle concerns, the items “Exercising and being physically active” and “Recent weight change (gain or loss)” had the highest loadings; corrected item-factor correlations ranged from 0.51 to 0.72. The exercise item and an item assessing body image concerns had the highest discrimination indices. The CSS-25 factors for health care team communication and relationship concerns are both comprised of 2 items each. These two 2-item factors, which continue to exhibit adequate factor loadings and item discrimination in the current EFA, are unmodified in the CSS-15+ scale. (Table 2).

**Table 2:** CancerSupportSource®: exploratory factor analysis of 25 items

Item	EFA of 25-item CancerSupportSource								
	F1	F2	F3	F4	F5	IDI	Factor #	Item–factor correlation	Action for short form
Emotional well-being									
Feeling nervous or afraid^†^	0.82					0.66	1	0.82	Retained
Feeling sad or depressed^‡^	0.77					0.77	1	0.82	Retained
Worrying about the future and what lies ahead^†^	0.77					0.85	1	0.81	Retained
Feeling lonely or isolated^‡^	0.71					0.71	1	0.81	Retained
Finding meaning and purpose in life	0.56					0.70	1	0.75	Dropped
Worrying about family, children, and/or friends	0.43					0.72	1	0.66	Dropped
Health insurance or money worries	0.39					0.69	1	0.60	Retained
Feeling irritable	0.34					0.66	1	0.65	Dropped
Symptom burden and impact									
Pain and/or physical discomfort		0.77				0.70	2	0.77	Retained
Moving around (walking, climbing stairs, lifting, etc.)		0.74				0.69	2	0.70	Dropped
Feeling too tired to do the things that you need or want to do		0.69				0.87	2	0.81	Retained
Managing side effects of treatment (nausea, swelling, etc.)		0.55				0.66	2	0.70	Dropped
Changes or disruptions in work, school, or home life		0.45				0.83	2	0.73	Retained
Thinking clearly (e.g., “chemo brain,” “brain fog”)		0.39				0.67	2	0.61	Retained
Sleep problems		0.30				0.70	2	0.59	Dropped
Transportation to treatment and appointments		0.30				0.30	2	0.51	Dropped
Body image and healthy lifestyle									
Exercising and being physically active			0.61			0.80	3	0.72	Retained
Recent weight change (gain or loss)			0.60			0.62	3	0.71	Dropped
Body image and feelings about how you look			0.53			0.73	3	0.69	Retained
Eating and nutrition			0.49			0.55	3	0.51	Dropped
Health care team communication									
Communicating with your doctor				0.48		0.47	4	0.58	Retained
Making a treatment decision				0.43		0.61	4	0.58	Retained
Relationships and intimacy									
Problems in your relationship with your spouse/partner					0.82	0.49	5	0.63	Retained
Intimacy, sexual function, and/or fertility					0.54	0.59	5	0.63	Retained
Additional items									
Tobacco or substance use—by you or someone in your household						0.16	N/A	N/A	Retained

^†^ indicates item is part of anxiety risk screening subscale; ^‡^ indicates item is part of depression risk screening subscale; *IDI* item discrimination index between upper and lower quartiles, based on total distress score

Judgmental item quality: three authors involved in the development of CSS-25 ranked each item from 1 (most relevant to short form) to 25 (least relevant to short form). An average rank was calculated for each item. The two items that make up the depression risk screening subscale and the two items that make up the anxiety risk screening subscale were the highest ranked items. Lowest ranked items included “Finding meaning and purpose in life,” “Feeling irritable,” and “Eating and nutrition”.

Scale reduction decisions: Criteria from all three indices were taken into consideration and used to sort items. Item reduction decisions were made, with preference given to items where all three quality indices were high. Scale refinement resulted in a 15-item short form of CSS plus a tobacco and substance use screener item (CSS-15+). At least two items from each of the five CSS-25 domains were retained to preserve multidimensionality, including five items from emotional well-being, four items from symptom burden and impact, two items from body and healthy lifestyle, two items from health care team communication, and two items from relationships and intimacy. All items from the anxiety and depression risk screening subscale items were retained (depression risk sensitivity = 91.4%, specificity = 79.5%; anxiety risk sensitivity = 91.8%, specificity = 70.9%) as previously described [14]. Additionally, the item about tobacco and substance use was retained due to clinical significance for risk assessment (Table 2).

### Phase II: corroboration of psychometric properties and dimensionality

#### Participants

Participant eligibility for Phase II was the same as Phase I of the study (see above) except for the date range: Phase II inclusion was limited to those 957 cancer patients and survivors who participated in the CER between January and December of 2017, were 18 years or older, lived in the USA, and had complete data for CSS-15+ and PROMIS items (used for validation analyses).

#### Measures

Procedures and measures were identical to Phase I of the study, with the exception that cancer-related distress was analyzed using the 15-item version of CSS plus the additional item assessing tobacco and substance use (CSS-15+), and 221 participants completed CSS again 30 - 90 min later to examine test-retest reliability. 

#### Analysis

Data analysis was again conducted using IBM SPSS Statistics 24.0 [19] and R 3.6.2 [20], with lavaan [21] and psych [22] R packages. To confirm dimensionality of the shortened scale, a confirmatory factor analysis was conducted using maximum likelihood factor extraction, fixing factor loadings for the first indicator in each factor to 1.0. Both absolute fit indices and relative fit indices were used to measure goodness of fit [23]. Internal consistency reliability was evaluated using Cronbach’s alpha. Test–retest reliability was measured with intraclass correlation coefficients. Nonparametric receiver operating characteristic (ROC) curve analyses were used to determine scoring thresholds for the 2-item CSS depression and anxiety risk scales, using PROMIS depression (*T* ≥ 60) and anxiety (*T* ≥ 62) scales as criterion scores [24, 25] as these correspond to conventional cutoffs for clinical risk significance using the PHQ-9 and GAD-7 legacy instruments [26–28]. Convergent validity was evaluated through Pearson correlations with PROMIS subscales. Discriminant validity was examined through the known-groups validation method, using Cohen’s *d* to estimate effect sizes between groups with the square root of the distress score.

#### Results

Participant socio-demographics: participants were predominantly female (67%), White (83%), and completed a college degree (56%). The average age was 58 years (range = 21–88), and average time since initial cancer diagnosis was 4.3 years (range = <1 to 49 years). The most commonly represented diagnoses included breast cancer (31%) and prostate cancers (16%; Table 1).

Confirmatory factor analysis: in confirmatory factor analysis, the model explained 59% of the variance and demonstrated good fit (RMSEA = 0.075, 90% CI = 0.069–0.082; SRMR = 0.033; CFI = 0.951; *χ*^2^(80) = 516.36, *p* < 0.001).

Internal consistency and test-retest reliability: Cronbach’s alpha for the full CSS-15+ scale was 0.94 (Table 3). Correlation between CSS-15+ and CSS-25 was 0.99, *p* < 0.001. The factors demonstrated moderate to large inter-correlations but were not redundant. Full scale test–retest reliability was .90, while individual factor ICCs were ≥ .79 (emotional well-being = .88; symptom burden and impact =.91; relationships and intimacy = .85; body image and healthy lifestyle = .79; health care team communication = .79).

**Table 3:** CSS-15+ scale and factor descriptive characteristics, inter-correlations, and internal reliability values (Cronbach’s *α*)

	# items	*M*/SD^†^	Inter-correlations					Cronbach’s *α*
			F1	F2	F3	F4	F5	
Total distress score (CSS-15)	15	16.27/13.48	0.95^*^	0.92^*^	0.80^*^	0.73^*^	0.73^*^	0.94
Total distress score + tobacco (CSS-15+)	16	16.58/13.82	0.94^*^	0.92^*^	0.80^*^	0.73^*^	0.73^*^	0.94
F1: Emotional well-being	5	1.13/1.05	–	0.81^*^	0.70^*^	0.65^*^	0.63^*^	0.89
F2: Symptom burden and impact	4	1.19/1.07		–	0.71^*^	0.60^*^	0.58^*^	0.87
F3: Body image and healthy lifestyle	2	1.44/1.02			–	0.50^*^	0.53^*^	0.54
F4: Health care team communication	2	0.66/0.96				–	0.43^*^	0.70
F5: Relationship and intimacy	2	0.84/1.07					–	0.69

^*^ denotes *p* < 0.001; ^†^mean/SD based on averaged factor scores, except for the total distress score, which is summed

Receiver operating characteristic curve analysis: using a PROMIS depression score of ≥ 60 to indicate risk for clinical levels of depression, a score of ≥ 3 on the 2-item CSS depression risk scale yielded a sensitivity and specificity of 81.5% and 84.8% (AUC = 0.888; Table 4). Using a PROMIS anxiety score of ≥ 62 to indicate risk for possible clinical levels of anxiety, a score of ≥ 3 on the 2-item CSS anxiety scale yielded a sensitivity and specificity of 82.9% and 74.8% (AUC = 0.865). Based on a cutoff score of 3 for each CSS risk scale, 30.2% of participants were at risk for clinically significant levels of depression and 40.3% were at risk for clinically significant levels of anxiety.

**Table 4:** Calculations of sensitivity and specificity for CancerSupportSource 2-item depression and 2-item anxiety risk scales

CSS risk score	PROMIS-29 comparison measure	EFA sample					CFA sample				
		% ≥ PROMIS threshold score	AUC	Cutoff	Sensitivity	Specificity	% ≥ PROMIS threshold score	AUC	Cutoff	Sensitivity	Specificity
Depression risk scale	Depression scale, *T* ≥ 60	19.2	0.923	2	97.0	63.2	22.6	0.888	2	89.8	68.0
				3	91.4	79.5			3	81.5	84.8
				4	79.4	89.3			4	73.2	91.8
Anxiety risk scale	Anxiety scale, *T* ≥ 62	21.7	0.903	2	98.0	48.2	26.2	0.865	2	89.2	57.4
				3	91.8	70.9			3	82.9	74.8
				4	81.3	85.2			4	73.3	86.0

*AUC* area under the curve. EFA ROC sample *n* = 1399 for depression risk; *n* = 1395 for anxiety risk; CFA ROC sample *n* = 957 for depression and anxiety risk

Convergent validity: CSS15+ total distress was moderately to strongly associated with all PROMIS subscales in the expected direction (*r*s = ± 0.50 to 0.74, *p*s < 0.001). Individual factors were moderately to strongly correlated with PROMIS subscales of similar concepts (Table 5). The CSS depression risk scale was strongly correlated with PROMIS depression (*r* = 0.78, *p* < 0.001) and the CSS anxiety risk scale with PROMIS anxiety (*r* = 0.71, *p* < 0.001).

**Table 5:** Pearson correlations between CSS-15+ and PROMIS validation measures

	PROMIS subscales						
	Depression	Anxiety	Social functioning	Physical functioning	Fatigue	Sleep disturbance	Pain interference
Total distress score (CSS-15)	0.74	0.67	−.56	− 0.58	0.59	0.50	0.65
Total distress score + Tobacco (CSS-15+)	0.74	0.67	− 0.55	− 0.58	0.59	0.50	0.65
F1: emotional well-being	0.77	0.71	− 0.49	− 0.50	0.54	0.48	0.57
F2: symptom burden and impact	0.66	0.59	− 0.63	− 0.65	0.66	0.50	0.72
F3: body image and healthy lifestyle	0.52	0.50	− 0.51	− 0.46	0.52	0.44	0.51
F4: health care team communication	0.48	0.45	− 0.33	−0.39	0.28	0.29	0.44
F5: relationships and intimacy	0.52	0.43	− 0.27	− 0.32	0.35	0.30	0.41
2-item depression risk scale	0.78	0.66	− 0.48	− 0.48	0.53	0.45	0.53
2-item anxiety risk scale	0.69	0.71	− 0.43	− 0.41	0.47	0.45	0.49

Values reported are Pearson correlation coefficients (*r*); all *p* < 0.001 for Pearson correlations

Discriminant validity: several group comparisons supported known-groups validity. The CSS-15+ total distress score was significantly (*t* = 3.29, *p* < 0.01) higher among those in active cancer treatment (*n* = 443) than among those who were not (*n* = 434, Cohen’s *d* = 0.22) and among those who were within 5 years of their cancer diagnosis (*n* = 629) than among those who were beyond 5 years (*n* = 242; *t* = 3.86, *p* < 0.001; Cohen’s *d* = 0.29), consistent with a small to medium value of *d*. Female participants reported more distress than male participants (Cohen’s *d* = 0.25; *t* = 3.64, *p* < 0.001). The square root of the total distress score was inversely associated with age (*r* = − 0.21, *p* < 0.001, *n* = 906) and time since diagnosis (*r* = − 0.11, *p* < 0.001, *n* = 871).

## Discussion

CancerSupportSource-15 (CSS-15+) is a reliable, valid, multidimensional distress screening tool that offers the benefits of the original 25-item CSS with a significant reduction in number of items. Notably, the total distress scores in CSS-15+ and the full-scale 25-item version of CSS approached nearly exact correspondence (*r* = 0.99), and internal consistency of the total distress score was similarly excellent in both tools. Despite its reduced length, CSS-15+ retains the same depression and anxiety risk scales as the 25-item CSS, and these scales demonstrated similarly high sensitivity coupled with adequate specificity [14]. Given the American Society of Clinical Oncology guidelines call for screening both depression and anxiety, retaining these clinical risk scales remains a valuable attribute of CSS-15+ [29].

Like the 25-item CSS, when implemented in oncology practice, CSS-15+ fulfills the American College of Surgeons Commission on Cancer patient-centered standards for distress screening and other quality and merit–incentive program standards (e.g., QOPI, OCM, and NCCN) [3, 6–8]. This shorter form will allow providers in busy community practices to quickly and efficiently identify patients who have clinically indicated levels of distress and/or specific unmet needs and, in full implementation, connect them with tailored resources, educational materials, supportive services, and referrals. Typical administration time for CSS-15+, including respondents indicating desired support resources for each item, ranges from 5 to 8 min. Given the recent focus on understanding barriers and facilitators of implementing screening practices [30], shorter length scales have the potential benefit of being a facilitator by increasing feasibility and acceptability to providers in busy settings [31]. Future research will examine CSS and CSS-15+ implementation in real-world clinical and community settings to determine how these tools can be optimally utilized to improve psychosocial and health outcomes [30].

One strength of CSS-15+ is its continued inclusion of two brief subscales that identify those individuals at risk for depression and/or anxiety. These items demonstrated good internal and external validity, and previously established cutoff scores [14] were reaffirmed in demonstrating both high sensitivity and adequate specificity. In screening for depression and anxiety, it is important to have high sensitivity to avoid missing those who may benefit from help. If patients meet one or both cut-off scores, we would recommend more detailed assessment of depression and anxiety be conducted to assist in the most appropriate triage. Additionally, CSS-15+ allows for flexible administration of the tobacco and substance use screening item, with its inclusion based on needs of the clinical setting. For consistency in research applications of the tool, we recommend calculating total distress based on the core 15 scale items.

Other strengths of this study include participation by a broad sample of survivors across diverse cancer care settings, diagnoses, and geographic regions. The study was well-powered [32], and decisions about item retention and removal were guided by systematic consideration of internal, external, and judgmental characteristics of items [17]. Limitations include self-selected samples of participants who are predominantly female, White, and well-educated, which may limit the generalizability of its findings with a more diverse socio-economic population. This sample is not representative of all cancer patients and survivors across the US, and there was greater representation of breast cancer patients, though it is representative of those who currently seek social and emotional support for cancer with a community-based cancer support organization. Given these programs were designed to detect distress and unmet needs and connect patients and survivors to free tailored resources and support services, the tool has potential for increasing behavioral health utilization among groups who historically have had less access to care [30]. We also used a validated measure of quality of life, PROMIS-29, to support examination of scale multidimensionality and convergent validity across the CSS-15+ factors; measurement of convergent validity for the relationships and intimacy factor was in part limited by a lack of PROMIS items focusing on partnered relationships.

Psychosocial distress screening is the necessary first step to comprehensively assessing and addressing cancer patient distress and unmet needs [33]. The multifactorial definition of psychosocial distress requires a multidimensional screening process that systematically examines patients’ concerns across key areas of the cancer experience, including emotional well-being, symptom burden and impact, body image and healthy lifestyle, health care team communication, and relationships and intimacy. Future work will include increased attention to the psychometric support for CSS and CSS-15+ within diverse populations across the cancer continuum, prioritizing how distress and unmet needs may vary based on individual trajectories of cancer care and survivorship. Additional research will also evaluate the facilitation of practical use of distress screening among health and supportive care teams, including rescreening, as well as the impact of distress screening on quality- and cost-related outcomes. Together, these efforts will support the provision of comprehensive, high quality, patient-centric cancer care.
